# How are automatic processes elicited by intended actions?

**DOI:** 10.3389/fpsyg.2013.00851

**Published:** 2013-11-13

**Authors:** Dana Ganor-Stern, Joseph Tzelgov, Nachshon Meiran

**Affiliations:** ^1^Department of Psychology, Achva Academic CollegeMP. Shikmim, Israel; ^2^Department of Psychology, Ben-Gurion University of the NegevBeer-Sheva, Israel

**Keywords:** automaticity, interference paradigm, task-dependence, automatic and intended actions

Although unintentionality is one of the key elements common to most traditional definitions of automaticity (e.g., Posner and Snyder, 1975; Shiffrin and Schneider, 1977; Hasher and Zacks, 1979; Jacoby et al., 1993; Tzelgov, 1997; Moors and De Houwer, 2006), more recent approaches pointed out that many automatic processes are affected and even elicited by intended processes (e.g., Bargh, 1994; Hommel, 2000; Tzelgov and Ganor-Stern, 2005; Yamaguchi and Proctor, 2012). However, there was no attempt to provide a systematic analysis of the different ways in which intended processes activate automatic ones. The present paper aims to fill this gap.

We view automatic processes as a product of interplay between information in long-term memory, reflecting life-long experience, and information in working memory, reflecting the requirement of the current situation. Such requirements are translated into task sets (Monsell et al., 2001), which activate the intended processes directly but may also activate some unintended processes indirectly. This activation of an unintended process (AUP) is a product of the overlap existing between the parameters of the intended process and those of unintended processes that are often highly practiced (Kornblum et al., 1990; Hommel, 2000).

Automatic processes can be triggered by AUP, and in some cases they will not start without it. Automatic processes differ in their dependence on AUP. Some automatic processes need very little AUP to occur, while for others, stronger AUP is needed (e.g., Bargh, 1989, 1992). Furthermore, AUP may not only trigger the occurrence of a process, but may also enhance its behavioral manifestation. For example, when the interference paradigm is used, the behavioral manifestation of the automatic processing, which is the interference effect, will be larger in conditions of strong vs. weak AUP.

We expand on the original taxonomy of dimensional overlap developed by Kornblum et al. (1990) and propose a classification of five levels of AUP, by which automatic processes are activated by intended ones. Furthermore, we demonstrate their effects using empirical findings of well-known indicators of automaticity. Note that a single task can provide AUP at multiple levels.

## AUP at the stimulus-location level

The experimental task directs participants' attention to an area in space or to an object (e.g., Bundesen, 1990; Scholl, 2001). Strong AUP at the stimulus-location level occurs when the task directs the participant's attention to the place where the automatically processed information is located. Consequentially, it encourages the processing of all dimensions of this object (e.g., Kahneman and Treisman, 1984; Kramer and Jacobson, 1991; Pratt and Hommel, 2003).

Findings with the Stroop task demonstrate AUP at this level. In this task, naming the color of the ink in which a color word is printed is hampered when the color word and the ink color are incongruent vs. congruent, indicating the automaticity of word reading (Stroop, 1935). Separating the word from the task-relevant colored stimulus presumably reduces the AUP, and thus results in a reduced Stroop effect relative to a situation where they are presented as an integrated object (e.g., Kahneman and Henik, 1981; MacLeod, 1998; Lamers and Roelofs, 2007). Additionally, spreading visual attention too widely by presenting another word in the display reduces the Stroop effect, an effect referred to as the dilution effect (Kahneman and Chajczyk, 1983). On the other hand, narrowing visual attention to the letter level by coloring only a single letter in a color word causes a reduction in the Stroop effect (Besner et al., 1997).

## AUP at the stimulus-identity level

The experimental task directs the participant's attention to a specific *stimulus*, which may be associated with one or more processes. Strong AUP at the stimulus-identity level exists when the task directs the participant's attention to a stimulus that is strongly associated with a particular highly practiced (yet, currently irrelevant) process. In such cases, attending to that stimulus might activate this process.

There is an especially strong association between words and the highly practiced skill of reading (Besner et al., 1997). As a consequence, attending to a word might activate the reading process and this should impair performance when the required task is not reading. Moreover, the activation of the reading process should increase with the resemblance of the stimulus to a word. Indeed, Monsell et al. (2001) showed that the slowing in a color naming task was dependent on the extent to which the non-words resembled words. Color naming was slower when the carrier stimulus resembled a word than when it did not. The difference between AUP at the stimulus location and at the stimulus identity level is similar to that between “where” and “what” in visual attention.

## AUP at the process level

The experimental task indicates the nature of the process required. There are two types of AUP at the process level. First, the intention to perform a process might activate related processes. Second, the process required by a task might still be active even when it is no longer needed.

### AUP from the process required by the current task

Experimental tasks require a wide variety of processes, such as naming, detection, classification, and comparison. The stronger the similarity or the association between the intended process and unintended ones is, the stronger the AUP. In this case, the process required by the task might activate related processes, perhaps through spreading activation.

Evidence for this type of AUP is provided, for example, by La Heij et al. (1998). Using the picture-word interference task, they found the gender congruency effect (difference in performance between trials in which the gender of the verbal title of the depicted object was congruent vs. incongruent with the gender of the word) only when participants produced noun phrases and not when they produced nouns only. Presumably the former activates gender-marking information to a greater extent than the latter does. Similar results were found in studies that looked at the effect of the grammatical category of the distractor word on performance in the same task (Pechmann and Zerbst, 2002; Pechmann et al., 2004). Analogous findings were reported by Vigliocco et al. (2005). Together, these studies suggest that the automatic activation of grammatical characteristics of an irrelevant stimulus during speech production depends on pre-activation of the specific grammatical information from the required task.

### AUP from the process required by the previous task

This type of AUP is strong when the unintended automatic process was required by the previous task. The processes required in a previous task, although not relevant anymore, might still be activated and influence the performance in the current task. The consistent pattern of poorer performance on task-switch trials (i.e., trials following a shift from one task to another) compared to task repetition trials is an example for such an effect (e.g., Kiesel et al., 2010; Meiran, 2010; Vandierendonck et al., 2010). It is partly due to carryover of activation from the previous task (e.g., Allport et al., 1994; Yeung et al., 2006).

## AUP at the stimulus-dimension level

The experimental task often requires a classification according to a certain dimension—size, color, polarity, etc. This might encourage the application of the same classification also to irrelevant stimuli. Strong AUP occurs when the dimension of interest is also applicable to other aspects or objects that are task-irrelevant (Kornblum et al., 1990; Kornblum and Lee, 1995).

Empirical evidence for this kind of AUP comes, for example, from the size congruency effect, which is found when a classification of digits according to physical size is required. Performance is enhanced when the numerical and physical sizes correspond, compared to when they do not, indicating the automatic processing of numerical magnitude (e.g., Henik and Tzelgov, 1982). In this case, the processing of the physical size required by the task might trigger the automatic processing of the irrelevant numerical size. Indeed, studies that used a luminance judgment found either no evidence for automatic numerical processing (Pinel et al., 2004) or a reduced effect (Rubinsten and Henik, 2005; Cohen Kadosh and Henik, 2006).

In a similar manner, the Stroop effect varies as a function of the overlap at the stimulus-dimension level between the relevant ink-color and irrelevant word. It is largest for color words, decreases for color-associated words, and decreases even more for words unrelated to color (Klein, 1964; MacLeod, 1991; Sharma and McKenna, 1998; Risko et al., 2006).

The effect of AUP at the stimulus-dimension level is also found for involuntary attention shifts. Folk et al. (1992) showed that the occurrence of involuntary shifts of attention depended on task demands and specifically, on the relation between the properties of the cue and the properties required to locate the target. They were present when the cue shared a feature property that was critical to the performance of the target task; for example, when the target was defined by color, colored cues produced involuntary attention shifts. Note that this AUP level differs from the previous one because AUP at the stimulus dimension refers to the stimulus dimension that is task-relevant, while AUP at the process level refers to the mental operation that is employed. Different processes might be applied to the same dimensions.

## AUP at the response level

The task set specifies the response categories and the way they are mapped onto the actual responses. Strong AUP occurs when the processing of the irrelevant dimension can be mapped onto the same set of responses specified for the relevant dimension. Such overlap may enhance the indications for automaticity. Indeed, larger Stroop effects are found for words that are part of the set of possible responses than for words that are not (Klein, 1964; MacLeod, 1991; Sharma and McKenna, 1998).

The present work could be viewed as an extension of Kornblum's dimensional overlap theory (Kornblum et al., 1990; Kornblum and Lee, 1995), which focused on stimulus-stimulus and stimulus-response overlap. We added to this framework the AUP at the stimulus location, stimulus identity, and process levels. Moreover, we related the systematic analysis of levels of AUP to degrees of automaticity. Automatic processes differ in the extent to which they depend on activation from the intended task, consistent with a gradual approach to automaticity (e.g., Moors and De Houwer, 2006). Some automatic processes need very little AUP to occur, with the least amount of AUP provided at the levels of stimulus location and stimulus identity. In fact, AUP at the stimulus level might be present even when there is no task at hand. In such cases, automatic processing will occur whenever the relevant stimulus is attended. In other cases, additional preconditions need to be met (e.g., Bargh, 1989, 1992). Such preconditions are provided by the other levels of AUP. The automatic process might occur only when the intended task involves a related process, a similar stimulus-dimension or the same response set.

## Summary

Although past works on automaticity acknowledged the fact that automatic processes are affected and even elicited by intended ones, there was no attempt to analyze this influence in detail. The present paper proposed a systematic analysis of the ways the intended process activates or elicits the occurrence of the automatic one. Examples for the effects of the AUP levels on automatic processes in different domains demonstrate the generality of this analysis. Results from other paradigms such as the flanker and Simon tasks could nicely fit into this analysis, however they were omitted from this paper, due to space limitations. Note that the different AUP levels are not mutually exclusive and a certain task might produce multiple levels of AUP to the automatic process. This is the case of the color-word Stroop effect, where all levels of AUP operate, and this may account for the robustness of the effect.

AUP affects not only the presence of the automatic process but also its behavioral indicators. As described in this paper, numerous studies from different domains showed that stronger AUP produced larger and stronger behavioral indicators of automaticity compared to weak AUP.
